# Outcome after thoracoscopic ventral stabilisation of thoracic and lumbar spine fractures

**DOI:** 10.1186/1752-2897-6-10

**Published:** 2012-10-16

**Authors:** Roman Pfeifer, Miguel Pishnamaz, Derek Dombroski, Nicole Heussen, Hans-Christoph Pape, Bernhard Schmidt-Rohlfing

**Affiliations:** 1 Department of Orthopaedic and Trauma Surgery, University of Aachen Medical Center, 30 Pauwels Street, Aachen, 52074, Germany; 2 Department of Orthopaedic Surgery, Parkland Health and Hospital Systems, Dallas, USA; 3 Department of Medical Statistics, RWTH Aachen University, Aachen, Germany

**Keywords:** Spine, Thoracoscopic surgery, Thoracolumbar fractures, Outcome

## Abstract

**Background and Purpose:**

Thoracoscopic-assisted ventral stabilisation for thoracolumbar fractures has been shown to be associated with decreased recovery time and less morbidity when compared with open procedures. However, there are a limited number of studies evaluating late clinical and radiological results after thoracoscopic spinal surgery.

**Methods:**

We performed an analysis of the late outcomes of thoracolumbar fractures after minimally invasive thoracoscopic ventral instrumentation. Between August 2003 and December 2008, 70 patients with thoracolumbar fractures (T5-L2) underwent ventral thoracoscopic stabilisation. Tricortical bone grafts, anterior plating systems (MACS-System), and cage implants were used for stabilisation. Outcomes measured include radiologic images (superior inferior endplate angle), Visual Analogue Scale (VAS), VAS Spine Score, quality of life scores SF-36 and Oswestry Disability Index (ODI).

**Results:**

Forty seven patients (67%, 47 out of 70) were recruited for the follow up evaluation (2.2 ± 1.5 years). Lower VAS Spine scores were calculated in patients with intra- or postoperative complications (44.7 (± 16.7) vs. 65.8 (± 24.5), p=0.0447). There was no difference in outcome between patients treated with bone graft vs. cage implants. Loss of correction was observed in both bone graft and titanium cage groups.

**Interpretation:**

The present study demonstrates diminished long-term quality of life in patients treated with thoracoscopic ventral spine when compared with the outcome of german reference population. In contrast to the other patients, those patients without intra-operative or post-operative complications were associated with improved outcome. The stabilisation method (bone graft versus spinal cage) did not affect the long-term clinical or radiographic results in this series.

## Introduction

Burst fractures are common thoracolumbar junction injuries [1]. Dorsal fixation of the thoracolumbar burst fractures is widely accepted as a treatment option [2,3]. Especially in unstable burst fractures, biomechanical investigations in vitro clearly support the reconstruction of the weight-bearing ventral column [4,5]. Anterior surgery may provide improved stability of the ventral column minimizing the possibility of secondary collapse and loss of correction [4-6]. Retrospective studies appear to demonstrate a reduction in morbidity associated with conventional thoracotomy for ventral stabilisation with comparable fusion rate in both open and minimally invasive surgical methods [7,8]. These studies have shown that the minimally invasive procedure may decrease the post-operative morbidity, allow early ambulation, and shorten the hospital stay [7,8]. The role of ventral fusion in treatment of these spine fractures is still unclear. Despite the advantages found in biomechanical studies, clinical investigations report inconsistent data in regard to maintaining correction [9-12]. In addition, there are only a few studies published evaluating late clinical and radiological results after minimally invasive thoracoscopic ventral instrumentation [9,13].

The objective of this retrospective analysis was to evaluate the long-term clinical and radiologic results after thoracoscopic ventral stabilisation of thoracic and lumbar spine fractures. Additionally, this study aimed to determine whether long-term quality of life in patients with ventral minimal-invasive approach would be comparable to a German representative population. Finally, we compared the outcome in patients treated with bone graft and those treated with titanium cage.

### Patients and methods

This investigation was designed as a single-centre (Level 1) retrospective cohort study. Patients were identified and prospectively subjected to standardised questionnaires. All patients included were treated between August 2003 and December 2008. Medical charts of the department of orthopaedic trauma surgery were screened for thoracic or lumbar spine fractures. Inclusion criteria were those patients with a fracture treated with ventral thorascopic fixation with either a tricortical bone graft alone, tricortical bone graft and stabilisation with either a modular anterior construct system (MACS-System®) (Aesculap AG & Co, Tuttlingen, Germany) or with a Telefix® Plate (Telefix, Synthes, Germany), isolated titanium cage implantation (Obelisk® cage; Ulrich, Ulm, Germany) with prior (at least partial) corpectomy, or a titanium cage and stabilisation with a MACS-System®. Plate implantation was predominantly used in those patients which had not received a prior dorsal fixation. Patients with paraplegia due to trauma, unavailable for follow-up or incomplete data were excluded.

### Data collection

Demographic and clinical data were obtained from the medical record. Data collected include demographic characteristics, mechanism of injury, type of injury, concomitant injuries, anatomical location of thoracolumbar fractures by radiographs and/or CT, fracture classification according to Magerl [14], type of surgical intervention, and postoperative complications. At follow-up, standardized questionnaires and scores addressing the quality of life, pain, patient satisfaction, sociodemographic characteristics and radiological measurements were addressed to patients. Prior verification of patients’ interest in participating in this study was obtained by phone. Radiological imaging was performed during routine post-operative examination.

### Assessment of outcome

Outcomes were assessed with the Visual Analogue Scale (0–10) prior to fracture stabilisation, one month after surgery and at the most recent follow-up. VAS Spine Score (range 0–100 points) was also used as this tool has been assessed and validated for outcome measurements in the treatment of patients with thoracolumbar injuries [15]. We also assessed patients’ satisfaction with a five choice satisfaction scale of “very satisfied,” “satisfied,” “neither satisfied nor dissatisfied,” “dissatisfied,” or “very dissatisfied”. Short-Form 36 (SF-36) was employed to assess overall quality of life; it includes an 8-item profile of functional and mental health summary measures [16]. Each item of the SF-36 score was compared with levels of representative German population (n=2.914) [17]. Oswestry Disability Index (ODI; range 0-100%), a validated outcome measure used in the management of spine disorders [18], was also recorded.

Radiographic assessment of correction was performed with the Superior-Inferior Endplate Angle (SIEA). The SIEA was supposed to measure correction after ventral stabilisation. Measurements were performed prior to the operation, one month after surgery and at follow-up (routine examination).

In order to identify risk factors for poor outcomes, patients were grouped and evaluated according to gender, age (≤ 50 years versus > 50 years), injury distribution (monosegmentalversus polysegmental), the presence or absence of concomitant injuries or intra-operative and/or post-operative complications.

### Statistics

Continuous variables are expressed as mean values ± standard deviations. Categorical data are presented by frequencies and percentage. For the comparison of the measured angles of the vertebral bodies at three different time points we performed a repeated measures analysis of variance (ANOVA) with type of surgery (cage vs. autologous graft) as grouping factor. Differences of VAS and VAS spine score at follow-up, ODI and SF-36 items between subgroups (defined above) were analyzed by means of t-tests. As all statistical tests were conducted solely in an explorative manner, no αlpha-adjustment for multiple testing was carried out. Thus, p-values of p ≤ 0.05 could be interpreted as statistically significant test results with respect to the investigated collective of this study.

Statistical analyses were carried out by the SAS statistical analysis software package (SAS for Windows, Version 9.1; SAS Institute, Cary, NC, USA).

### Ethics

This study was performed in accordance with the ethical standards of the responsible committee on human experimentation and with the Helsinki Declaration of 1975, as revised in 2000.

## Results

**Figure 1:**
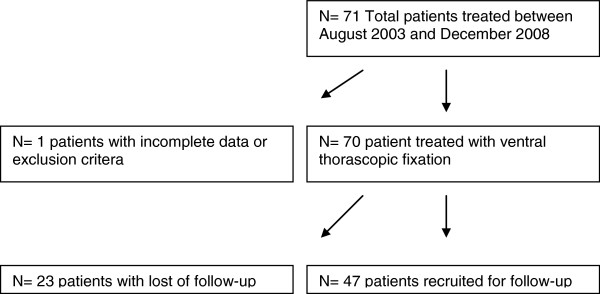
Flow-chart demonstrating the patient recruitment.

During the observation period (August 2003 to December 2008), 70 trauma patients with thoracolumbar injuries were admitted to our trauma center and underwent ventral thoracoscopic fracture stabilisation (flowchart of patients recruitment Figure 1). Mean patient age on admission was 46 years (± 14; range 15–74 years), and 65.7% (n=46) were male and 34.3% (n=24) female. The follow up was 2.2 years (± 1.5 years). Causes of injury are shown in Table 1. Falls (46%) and road traffic accidents (26%) were the most common causes of traumatic thoracolumbar injuries. Fracture classification according to Margerl’s definition, frequency and distribution of thoracolumbar fractures are summarized in Table 1. The majority of patients sustained isolated vertebral body fractures at the thoracolumbar junction (L1 and Th12). Two or more vertebral body fractures were observed in 37.1% of patients. Associated injuries were predominantly injuries of the chest (31%), head (12.7%), upper and lower extremities including pelvis (15.5%), and blunt abdominal trauma (4.2%). Approximately 50% of our study population sustained isolated thoracic and lumbar fractures without additional injuries in other regions.

**Table 1:** Cause of Injury and Demographic Data

**Parameter**	**Patients total N = 70**	**Pat. at follow up N = 47**
Age at surgery (yr ± SD)	45 (14)	45 (14)
Male (N (%))	46 (65)	29 (61)
Mean Follow Up (years ±SD)	--	2.2 (1.5)
**Mech. of Injury**	**N (%)**	**N (%)**
Road Traffic Accident	18 (26)	12 (26)
Fall from height	32 (46)	23 (49)
Accident at Work	7 (10)	5 (11)
Sports Accident	6 (9)	4 (9)
Pathologic Fracture	2 (3)	2 (4)
Other	5 (7)	1 (2)
Total	70 (100)	47 (100)
**Fracture Localisation**	**(%)**	**(%)**
Th5	3.2	-
Th6	3.2	2.1
Th7	6.4	8.5
Th8	5.5	-
Th9	2.2	2.1
Th10	4.4	2.1
Th11	8.8	10.6
Th12	25.3	21.3
L1	35.2	48.9
L2	5.2	4.2
**Fracture Classification**	**(%)**	**(%)**
A1	12.9	12.7
A2	7.1	8.5
A3	70	65.9
B2	2.9	4.2
B3	1.4	-
C1	2.9	2.1
C2	1.4	-
unknown	5.7	6.3

### Operative interventions and associated complications

In 90% (n= 63) of patients dorsal instrumentation with a posterior internal fixator was performed prior to ventral procedure. The remaining 10% of patients were treated with anterior fusion alone. In 68.6% of cases monosegmental fixation was used. Polysegmentalstabilisation was necessary in 31.4%. Incomplete burst fractures were treated by monosegmental ventral stabilisation. In complete burst fractures a bisegmental procedure was chosen. The majority (n=36, 51.4%) of the 70 patients were treated with tricortical bone graft alone. Patients (17.1% (n=12)) received an additional stabilisation with a plate when prior dorsal internal fixation was not performed. Isolated titanium cage implantation was performed in 17.1% (n=12). Cage implantation with additional plate stabilisation was performed in 14.1% (n=10) patients.

Complications were observed in 31.4% (n=22) of our study population. The most common intra-operative complications were iatrogenic fractures of ribs (13.6%, n= 3), followed by intercostal nerve irritation (9%, n=2) and vascular injuries (4.5%, n=1) and. Post-operative complications included tension pneumothorax (13.6%, n=3), atelectasis (13.6%, n=3), and systemic infections (pneumonia) (13.6%, n=3). No severe complications were observed in our study population (e.g. aortic and spleen injuries, severe wound infections).

### Radiological outcome at follow up

**Figure 2:**
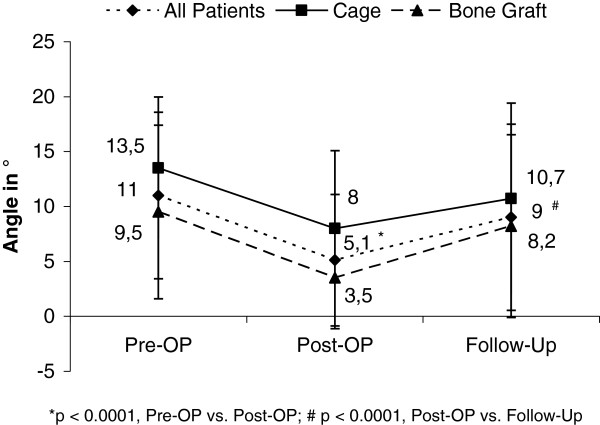
Superior-Inferior Endplate Angle (SIEA) measured preoperatively, immediately postoperatively and at follow up.

Radiological evaluation of the superior-inferior endplate angle (SIEA) is shown in Figure 2 (66 out of 70, patients with complete radiological imaging). The mean preoperative SIEA was 11.0°±7.5 in all patients. Patients who underwent ventral fusion with a spinal cage demonstrated a higher SIEA when compared to patients stabilised with bone graft (13.5°±6.5 versus 9.5°±7.9, p=0.0969). Marked reduction of the SIEA (in mean 5.9°) was measured postoperatively in our study population (11.0°±7.5 versus 5.1°±5.9; p<0.0001). At follow up, the mean increase of the SIEA was 3.9° in all patients (5.1°±5.9 postoperatively versus 9.0 °± 8.5; , p<.0001).

### Clinical outcome

#### Pain and Patients Satisfaction

The mean intensity of pain according to VAS obtained prior to surgical intervention was 5.4 (± 3.6). Pain level increased (5.6 ± 2.3) within the first month after surgery and then clearly decreased (3.6 ± 2.3) at the time of follow up examination. Analysis of patient’s satisfaction revealed 68% (n=32) were satisfied or very satisfied with their operative reconstruction. Less satisfied patients (n=1 very dissatisfied, n=2 dissatisfied) were more frequently subjected to revisions or re-operations. Revisions (n=3) were performed due to local wound infection (n=1), hardware failure (n=1) and implant malposition (n=1).

Patients without concomitant injuries (3.2 (±2.2) versus 4.1 (±2.6); p=0.1734) or intra- and postoperative complications (3.1 (±2.4) versus 4.8 (±1.8); p= 0.0720) were shown to have less pain. However, statistical analysis did not show these trends to be significant. VAS Spine Score analysis showed patients without associated complications had significantly superior score levels (65.8 (±24.5) versus 44.7 (±16.7); p=0.0447). In addition, younger patients (≤ 50 years) and individuals without associated injuries also demonstrated superior pain results. However, neither group reached statistical significance (Table 2).

**Table 2:** Outcome analysis at follow up

	**Parameter**	**N (%)**	**VAS at Follow-Up**	**VAS Spine Score at Follow-Up**	**ODI**	**SF-36 physical**	**SF-36 mental**
**All Patients**		47(100)	Mean 3.6 (SD±2.3) 95%CI [2.9-4.3]	Mean 59.9 (SD±24.4) 95%CI [52.9-66.9]	Mean 22.9 (SD±19.1) 95%CI [17.5-28.4]	Mean 40.4 (SD±11.1) 95%CI [37.2-43.5]	Mean 52.6 (SD±3.4) 95%CI [51.6-53.6]
**Sex**	Male	29(61.7)	Mean 3.5 (SD±2.4) 95%CI [2.8-4.2]	Mean 58.7 (SD±25.8) 95%CI [55.3-66.1]	Mean 24.6 (SD±20) 95%CI [18.8-30.3]	Mean 40.3 (SD±12.2) 95%CI [36.8-43.7]	Mean 52.6 (SD±3.6) 95%CI [51.5-53.6]
	Female	18(38.3)	Mean 3.7 (SD±2.3) 95%CI [3.1-4.4]	Mean 61.9 (SD±22.3) 95%CI [55.5-68.3]	Mean 20.4 (SD±17.6) 95%CI [15.4-25.5]	Mean 40.5 (SD±9.6) 95%CI [37.7-43.2]	Mean 52.7 (SD±3.2) 95%CI [51.8-53.6]
**Age**	≤ 50 years	27(57.4)	Mean 3.4 (SD±2.5) 95%CI [2.6-4.1]	Mean 63.1 Median 63.9 95%CI [55.1-71.0]	Mean 20.7 Median 16 95%CI [15.1-26.4]	Mean 41,8 Median 41.6 95%CI [38.6-44.9]	Mean 52.2 Median 51.1 95%CI [51.1-53.2]
	> 50 years	20(42.6)	Mean 3.9 (SD±2.2) 95%CI [3.3-4.6]	Mean 55.7 SD±18.7) 95%CI [50.4-61.1]	Mean 26 (SD±18.2) 95%CI [20.8-31.2]	Mean 38.4 (SD±11.1) 95%CI [35.2-41.6]	Mean 53.2 (SD±3.0) 95%CI [52.7-54.1]
**Injury Distribution**	Monosegmental	33(70.2)	Mean 3.7 (SD±2.2) 95%CI [3.0-4.3]	Mean 58.2 (SD±22.0) 95%CI [51.9-64.5]	Mean 23.3 (SD±18.1) 95%CI [18.1-28.5]	Mean 39.9 (SD±10.4) 95%CI [36.9-42.8]	Mean 52.7 (SD±3.1) 95%CI [51.8-53.6]
	Polysegmental	14(29.8)	Mean 3.5 (SD±2.8) 95%CI [2.7-4.2]	Mean 64.0 (SD±29.7) 95%CI [55.5-72.5]	Mean 22.3 (SD±21.9) 95%CI [16.1-28.5]	Mean 41.5 (SD±13.1) 95%CI [37.8-45.3]	Mean 52.5 (SD±4.2) 95%CI [51.3-53.7]
**Treatment**	Bone Graft	31(66)	Mean 3.7 (SD±2.1) 95%CI [3.1-4.3]	Mean 59.4 (SD±23.2) 95%CI [52.7-66.1]	Mean 24.3 (SD±17.6) 95%CI [19.3-29.4]	Mean 38.7 (SD±10.8) 95%CI [35.7-41.9]	Mean 52.6 (SD±3.1) 95%CI [51.7-53.5]
	Spinal Cage	16(34)	Mean 3.4 (SD±2.8) 95%CI [2.6-4.2]	Mean 60.9 (SD±27.2) 95%CI [53.2-68.8]	Mean 20.4 (SD±22) 95%CI [14.1-26.5]	Mean 43.4 (SD±11.4) 95%CI [40.2-46.7]	Mean 52.7 (SD±3.4) 95%CI [51.5-53.9]
**Concomitant Injuries**	No	29(61.7)	Mean 3.2 (SD±2.2) 95%CI [2.6-3.9]	Mean 63.4 (SD±22.1) 95%CI [57.0-69.7]	Mean 21.8 (SD±18.5) 95%CI [16.5-27.1]	Mean 42.7 (SD±9.15) 95%CI [40.1-45.3]	Mean 52.0 (SD±3.4) 95%CI [51.1-52.9]
	Yes	18(38.3)	Mean 4.1 (SD±2.6) 95%CI [3.4-4.9]	Mean 54.5 (SD±27.4) 95%CI [46.6-62.3]	Mean 24.9 (SD±20.3) 95%CI [19.1-30.7]	Mean 36.6 (SD±13.2) 95%CI [32.8-40.4]	Mean 53.6 (SD±3.4) 95%CI [52.6-54.6]
**Complications**	No	34(72.3)	Mean 3.1 (SD±2.4) 95%CI [2.4-3.8]	Mean 65.8 (SD±24.5)* 95%CI [58.8-72.8]	Mean19.8 (SD±18.7) 95%CI [14.4-25.1]	Mean 42.6 (SD±11.7) 95%CI [39.4-45.8]	Mean 51.7 (SD±3.4) 95%CI [50.7-52.8]
	Yes	13(27.7)	Mean 4.8 (SD±1.8) 95%CI [4.3-5.3]	Mean 44.7 (SD±16.7) 95%CI [39.9-49.5]	Mean 31.4 (SD±18.1) 95%CI [26.2-36.6]	Mean 34.6 (SD±9.09) 95%CI [32–37.2]	Mean 54.9 (SD±2.3) 95%CI [54.2-55.5]

*p < 0.05 / 95% CI = confidence interval / SD = standard deviation.

#### Quality of life

In order to evaluate the quality of life after thoracoscopic ventral stabilisation of thoracolumbar spine fractures, ODI and SF-36 scores were measured at follow up (Table 2). According to ODI scores, intra- and postoperative complications affected the quality life after ventral thoracoscopic fusion. Patients without associated complications demonstrated superior ODI scores (19.8 (±18.7) versus 31.4 (±18.1); p= 0.1207). Moreover, individuals with 50 years of age and younger demonstrated improved quality of life (20.7 (±19.7) versus 26 (±18.2); p= 0.5615) as well; however, this difference did not reach statistical significance.

**Figure 3:**
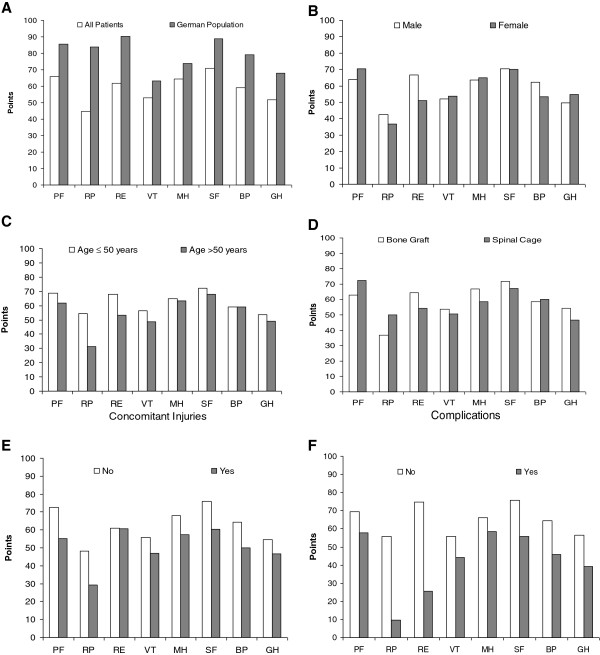
**Mean results of patients’ response to the SF-36 analysis at follow up.** (**A**) shows the comparison against the average of the representative German population (n=2.914). Moreover, study population was evaluated according to the gender (**B**), age (**C**), type of treatment (**D**), and the presence of concomitant injuries (**E**) or intra-operative and/or post-operative complications (**F**). PF, physical functioning; RP, role physical; RE, role emotional; VT, vitality; MH, mental health; SF, social functioning; BP, bodily pain; GH, general health.

All eight items of the SF-36 score in our study population (SP) were markedly below levels recorded in a representative German population (GP) (Figure 3 A-F). In particular, both Role-Physical (RP) and Role-Emotional (RE) items representing role limitations because of physical health and emotional problems, respectively, showed high score differences. Physical health related role limitations were mainly problematic in patients older than 50 years of age (RP: ≤50 54.6 (± 45.9) versus >50 M 31.3 (± 40.5)). Moreover, patients who sustained intra- and postoperative complications and/or concomitant injuries scored much lower. Deteriorations of the physical functioning and related role limitations were found in these groups (RP: with complications 9.6 (± 19.2) versus no complications 55.7 (± 44.3)).

## Discussion

Kyphosis recurrence and chronic pain has been reported in patients with fractures of the thoracolumbar spine when treated with dorsal instrumentation only [19,20]. Due to these finding it has been suggested that an additional ventral fusion may improve outcomes [21]. Endoscopic techniques for ventral fixation appear to have advantages in regard to pain and early recovery [22,23]. Moreover, the duration of administration of pain medication and overall dose was reported to be lower in comparison with patients subjected to open surgery [22,23].

Our main findings can be summarised as follows: (1) Intra-operative and post-operative complications significantly affected the long-term pain levels (VAS Spine Score). (2) Despite the treatment efforts, the quality of life (SF-36) in our study group was markedly below the levels reported in representative German population. (3) Loss of correction as measured by SIEA appears to take place in both bone allograft and titanium cage groups.

The following limitations have to be considered when interpreting the results of our study. The retrospective acquisition of data and the limited patient number represent a limitation of this investigation. Nevertheless, we feel that the relatively long observation period (follow-up 2.2 years) and standardised outcome assessment (pain and quality of life) represent the main strength of this work.

In our series, pain intensity increased directly after operation and decreased at follow up. Our findings are in line with comparable investigations analysing pain intensity after ventral fixation [11]. A recently published randomized prospective study analysing the long-term (minimum 4 years) clinical and radiographic outcome after anterior-only stabilisation plating with bone autograft (n= 32) versus titanium mesh cage (n= 33) of thoracolumbar burst fractures showed low average VAS scores for back pain in both groups at follow up (autograft 1.6 ± 0.7 versus cage 1.2 ± 1.1) [9]. In comparison to the previous study, higher VAS scores (mean 3.6 ± 2.3) were measured at follow up in the present analysis. We believe that the inclusion of a younger study population (all below 58 years) with less severe spine injuries (all monosegmental) was mainly responsible for superior results in this particular study. A lower incidence of osteoporosis, fewer co-morbidities, and improved bone healing are possible factors associated with improved outcomes in younger patients. Moreover, our investigation has shown that patients who sustained polysegmental fractures were also associated with less pain by the VAS scoring system. These findings may be explained by the fact that patients with polysegmental fractures were mainly subjected to fracture fixation using spinal cage. In our study, the spinal cage group also demonstrated superior VAS levels; however, statistical significance was not reached. The presence of donor site pain has been pointed out by Dai and co-authors in patients with iliac crest bone autograft for anterior-only stabilisation of thoracolumbal burst fractures [9]. Donor site morbidity was still present in 26 (82.3%, follow up minimum 4 years) patients with iliac crest bone graft harvest.

The VAS Spine Score values of 58.25 points (± 22.19) after operation and 66.08 points (± 25.03) at follow up (23 month) were reported in 53 patients (mean age 43 years, range 19 to 68) with thoracolumbar spine injuries, all treated with combined posterior-anterior stabilisation and fusion [15]. In a healthy reference population (n=136) average VAS Spine Scores were 91.95 points (±7.25) [15]. VAS Spine Scores of 59.9 points (± 24.4) were measured at follow-up in our study population. These findings are similar to the results demonstrated by Knop et al. [15]. Moreover, the absence of intra- and postoperative complications was associated with improved VAS Spine Score outcome in our study. Approximately 27.1% of our study population experienced intra- or postoperative complications. Common intra-operative complications were fractures of adjacent ribs and neurovascular structures and postoperative complications include pulmonary dysfunction due to pneumothorax, atelectasis or infections (pneumonia). A prospective, multicenter study analysing the operative treatment of thoracolumbar spine fractures revealed a complication rate of 29.7% in patients who underwent anterior approach alone and 13.7% after combined posterior-anterior surgery [24]. This study and others reported a comparable spectrum of associated complications [11,22,24,25].

Multiple studies have demonstrated loss of correction after isolated dorsal instrumentation of thoracolumbar fractures [19-21]. Thereupon, authors hypothesized ventral column fixation would be biomechanically superior in maintaining sagittal alignment [21]. However, secondary loss of correction was also observed within the first year of those who underwent posterior stabilisation followed by anterior fusion [11]. The collapse of the vertebral body appears to take place even after anterior-posterior stabilisation. Moreover, some authors believe cage implantation might be superior to allograft in regard to maintenance of correction [26]. Successful long-term (at least 4 years) maintenance of kyphotic correction was demonstrated by Dai et al. in patient with burst fractures treated with anterior only spinal cage or bone graft [9]. The authors concluded that ventral fusion is feasible to eliminate collapse after burst fractures. Our analysis shows no differences between the treatment methods (cage versus allograft). However, a significant increase of superior-inferior-endplate angle at follow up was observed in both treatment methods.

Quality of life is an important assessment to evaluate patient-oriented treatment success after surgical intervention. Chronic pain, functional disabilities, mental health and socio-economic parameters (e.g. employment status) are among the most important factors influencing the long-term life satisfaction after trauma [13,27,28]. Numerous studies have used the SF-36 score for overall health and quality of life assessment in patients with spine disorders [13,19,27,28]. Using this score, Briem and co-authors reported an impaired quality of life in patients (follow up 5.3 years, ± 1.7 years) with thoracolumbar spine fractures when compared to healthy controls [28]. Our investigation agrees with these findings demonstrating marked impairments of SF-36 in comparison to a representative German population (Figure 3 A-F). However, in comparison to our study, younger patients (< 65 years) with less severe spine injuries (63.9% were treated non-operatively) were more common in Briem’s study. Long-term (16.3 years) quality of life after conservatively treated thoracolumbar fractures as measured by SF-36 was comparable to scores reported in patients with chronic back pain [29]. Others found similarities to patients with other chronic diseases (dialysis [30] or diabetes [31]).

Thoracoscopic surgical intervention within the thoracic cavity was associated with typical procedure-related pulmonary complications (pleural effusion, pneumothorax, pneumonia, atelectasis) in this series. The presence of these complications may interfere with the direct post-operative recovery. However, long-term consequences due to these adverse events are unlikely. Out of 19 patients who experienced a complication, 3 patients demonstrated a complication (n=1 spinal cord injury, n=2 nerve injury) which might potentially have a long-term impact on functional outcome and/or quality of life. Moreover, reoperation also negatively influences patient’s satisfaction at follow up. All patients who were subjected to operative revision were dissatisfied or very dissatisfied with their outcome. Whether lasting disability, chronic pain, dissatisfaction due to the complication itself or other unrelated factors mainly affected the long-term outcome (ODI) of patients with complications unfortunately cannot be extracted out of our data.

## Conclusion

The present study demonstrates diminished long-term (2.2 years) quality of life (SF-36) in patients treated with thoracoscopic ventral spine stabilisation either with bone graft or spinal cage. However, some patients appear to demonstrate superior results. Patients without intra-operative or post-operative complications were associated with significant improved pain outcome (VAS Spine Score). Moreover, the stabilisation method (bone graft versus spinal cage) did not influence the long-term clinical or radiographic results in this series. Further prospective clinical studies and biomechanical in vitro investigations are necessary to identify the factors affecting the bone fusion and loss of correction in patients with thoracic burst and lumbar fractures. In addition, further studies should be performed to evaluate treatment strategies and improve quality of life.

## Competing interests

The authors declare that they have no competing interest.

## Authors’ contributions

All authors were involved in the research project and preparation of the manuscript. BSR and HCP made substantial contribution to the conception and design, and gave a critical and final approval. RP, MP, and DD have collected the data and made an analysis and interpretation of these data. They also made a draft of the manuscript and revisions. NH performed the statistical analysis and critical statistical evaluation of the data. All authors read and approved the final version of the manuscript.
